# A Novel Metal Foam‐Supported Solid Oxide Fuel Cell With High Specific Power

**DOI:** 10.1002/advs.202517694

**Published:** 2026-02-12

**Authors:** Jingbo Ma, Ying Yang, Meng Ni, Yanxiang Zhang, Mufu Yan

**Affiliations:** ^1^ School of Materials Science and Engineering Harbin Institute of Technology Harbin China; ^2^ China Academy of Machinery Beijing Research Institute of Mechanical & Electrical Technology Co., Ltd. Beijing China; ^3^ Department of Building Environment and Energy Engineering Research Institute for Sustainable Urban Development & Research Institute for Smart Energy The Hong Kong Polytechnic University Hong Kong China

**Keywords:** impact resistance, lightweight, metal foam support, solid oxide fuel cells, specific power

## Abstract

Solid oxide fuel cells (SOFCs) have emerged as a promising energy conversion technology for transportation applications, including aerospace, maritime, and new energy vehicles, owing to high energy conversion efficiency, high energy density, reliability, fuel flexibility, and environmental friendliness. However, the low specific power of SOFCs hinders their commercialization for mobile power applications. To develop lightweight SOFCs, this study presents a novel SOFC structure utilizing a highly porous, large‐pore metal foam as the support. By employing commercially available functional layer materials and established fabrication processes, the proposed SOFC achieves a peak power density of 1.01 W cm^−^^2^ at 650°C, with a specific power of 6.56 kW kg^−1^, representing a several‐fold improvement over state‐of‐the‐art design. Additionally, its impact resistance is improved by more than tenfold compared to conventional anode‐supported SOFCs. This breakthrough is attributed to the metal foam support, which retains a high porosity of 90% after sintering, as revealed by 3D computed tomography. The enhanced effective diffusion coefficient (0.75) significantly reduces concentration polarization. Moreover, the metal foam support reduces the areal mass of the SOFC to 0.15 g cm^−^^2^, synergistically improving the specific power and better meeting the demands of mobile power systems for high specific power and impact resistance.

## Graphical abstract

Solid oxide fuel cells (SOFCs) employing metal foam supports with an ultrahigh porosity of 90% and average pore size of 100 µm are successfully fabricated. The metal foam architecture significantly enhances gas diffusion coefficients, effectively reducing concentration polarization while simultaneously decreasing areal mass. Remarkably, the optimized cells demonstrate a remarkable specific power density of 6.56 kW kg^−1^.

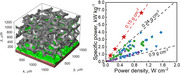

## 1 Introduction

The ongoing advancement of economic globalization has accelerated the transportation sector's growth, driving escalating demand for mobile power solutions. As an efficient and environmentally friendly energy conversion technology, solid oxide fuel cells (SOFCs) have attracted significant attention in the era of energy transition [1]. The high fuel flexibility and operational reliability of SOFC systems make them highly competitive for mobile power applications, spurring extensive R&D in fields such as aviation, aerospace, marine transportation, and new energy vehicles [2, 3, 4, 5, 6, 7, 8, 9].

**FIGURE 1:**
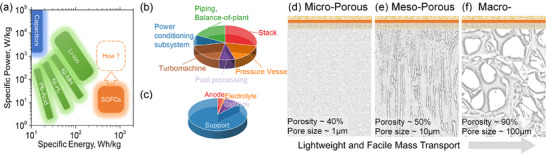
(a) Ragone plot. (b)Mass distribution of different components in a typical SOFC‐APU system. (c) Typical mass distribution of components in repeating SOFC functional units. Schematic illustrations of (d) micro‐porous, (e) meso‐porous, and (f) macro‐porous support architectures.

It is well‐established that the mass and volume of fuel cell systems are critical limiting factors for their commercialization in mobile power applications. Increasing the specific power could significantly reduce the payback time [4, 10, 11]. However, as illustrated in Figure 1a, SOFCs maintain relatively low specific power compared to competing energy technologies. In the automotive sector, current SOFC systems deliver only 0.4 kW kg^−1^, failing to meet the 0.65 kW kg^−1^ industry benchmark. More critically, S. Hashimoto et al. reported an SOFC system for aircraft applications with a specific power of merely 0.14 kW kg^−1^, which is substantially lower than NASA's 1.0 kW kg^−1^ operational requirement [12, 13, 14]. This pronounced performance gap unambiguously confirms that specific power enhancement constitutes the fundamental requirement for SOFC deployment in mobile power systems.

In auxiliary power units (APUs), SOFC stacks account for over 20% of the total mass budget. Figure 1b illustrates the typical mass distribution of an SOFC‐APU system [4]. NASA studies have demonstrated that interconnect mass distribution exhibits topology dependence. Notably, support architectures persistently dominate the mass allocation (≥80%) within the SOFC repeating unit, as shown in Figure 1c [15]. Consequently, minimizing the support structure mass while preserving power density represents the most critical optimization strategy for achieving substantial specific power improvements in SOFC systems.

To enhance the areal power density, SOFC configurations have transitioned from electrolyte‐supported to anode‐supported designs [16]. Wachsman et al. demonstrated that incorporating advanced functional layers on anode supports can achieve exceptional peak power densities of ∼2 W cm^−^^2^ at 650°C [17]. However, these configurations suffer from excessive mass and elevated polarization losses due to their thick ceramic support layers. This limitation has been addressed through the development of third‐generation metal‐supported SOFCs, which enable dramatic reductions in ceramic layer thickness. By substantially decreasing total cell mass while simultaneously reducing ohmic losses and mitigating concentration polarization, this architectural innovation significantly enhances electrochemical performance and potentially boosts specific power. Metal‐supported SOFCs offer additional advantages over conventional all‐ceramic structures, including superior mechanical strength and enhanced sealing capability, making them particularly suitable for mobile applications where durability and reliability are paramount [16].

Current SOFC support structures are mainly fabricated as microporous or straight‐pore configurations via powder sintering or freeze‐drying, typically attaining porosities of ∼40% and ∼60%, respectively, depending on material properties and processing conditions [18, 19, 20, 21]. For applications demanding ultralightweight designs, metal foams with higher porosity and larger pore sizes offer a promising alternative, potentially reducing both support density and gas transport resistance, as illustrated in Figure 1d–f. While theoretically ideal, this design's practical implementation faces significant challenges. First, metal foams coarsen during high‐temperature sintering of ceramic functional layers due to their limited thermal stability [22]. Second, conventional fabrication methods, such as tape casting, spin coating, or screen printing, cannot consistently produce defect‐free, uniform functional layers on large‐pore substrates. Visvanichkul et al. attempted foam compaction to address this issue, but this approach inevitably reduced porosity, although exact values were not provided [23]. Gondolini et al. used a pre‐oxidized NiCrAl foam with a 50‐µm‐thick transition layer deposited via stencil printing to cover large pores and reduce surface roughness, enabling subsequent screen printing of the anode functional layer [24]. However, such modifications compromise the inherent advantages of metal foams, contradicting our design goals.

Therefore, this work employs a 90%‐porosity metal foam as the substrate for SOFC fabrication to maximize weight reduction. To simultaneously maintain the metal foam's initial architecture and establish robust interfaces with functional layers, a novel fabrication route for metal foam‐supported SOFCs (MF‐SOFCs) was developed. The fabrication process began with the step‐by‐step preparation of Ni‐Gd_0.1_Ce_0.9_O_1.95_ (GDC) anode, GDC electrolyte, and La_0.6_Sr_0.4_Co_0.2_Fe_0.8_O_3‐δ_ (LSCF)‐GDC cathode functional layers via tape casting. A Fe‐Ni (1:1 mass ratio) metal foam support was selected after rigorous evaluation of chemical compatibility and thermal expansion coefficient matching. To ensure optimal bonding, an interfacial layer with identical composition was precisely deposited to connect the metal foam support with the functional layers. The assembled structure was finally co‐sintered at temperatures significantly lower than conventional ceramic sintering temperatures, producing the completed MF‐SOFC. The as‐fabricated MF‐SOFC exhibited exceptional performance, delivering a peak power density of 1.01 W cm^−2^ at 650°C under humidified hydrogen atmosphere. Remarkably, the specific power attained 6.56 kW kg^−1^, which validates the feasibility of the fabrication approach. These results constitute a major advancement in lightweight SOFC technology.

## 2 Results and Discussion

### 2.1 Electrochemical Performance Evaluation of MF‐SOFCs

**FIGURE 2:**
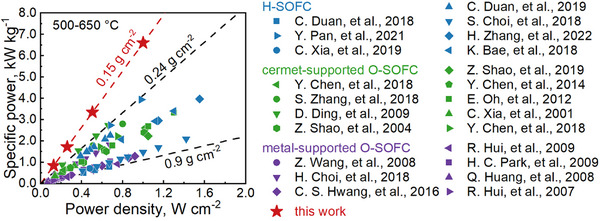
Comparison of specific power vs. peak power density between the present work and literature‐reported SOFCs, including H‐SOFCs, cermet‐supported O‐SOFCs, and metal‐supported O‐SOFCs.

The MF‐SOFCs developed in this work achieve a breakthrough with a 90% porosity support structure, yielding an areal mass of just 0.15 g cm^−^^2^—at least 37.5% lower than prior SOFC reports. This architecture delivers a specific power of 6.56 kW kg^−1^ at 650°C in a 3 vol% H_2_O/H_2_ atmosphere, outperforming existing technologies by several‐fold to an order of magnitude. Figure 2 compares the specific power outputs of intermediate‐temperature SOFCs reported over the past two decades, including proton‐conducting SOFCs (H‐SOFCs), cermet‐supported oxygen‐ion conducting SOFCs (O‐SOFCs), and metal‐supported O‐SOFCs [20, 25, 26, 27, 28, 29, 30, 31, 32, 33, 34, 35, 36, 37, 38, 39, 40, 41, 42, 43, 44, 45, 46]. The MF‐SOFC demonstrates superior specific power in the range of 500–650°C compared to metal‐supported SOFC technologies. At 600–650°C, the MF‐SOFC achieves areal power densities comparable to those of H‐SOFCs while far exceeding those of cermet‐supported O‐SOFCs (Figure S1). This superior specific power stems from the synergistic combination of exceptionally low areal mass and high areal power density.

MF‐SOFCs were fabricated using conventional functional layer materials and tape‐casting techniques. Anode‐supported SOFCs (referred to as Con‐SOFCs) were fabricated as controls to systematically assess the effects of metal foam support on electrochemical performance. Con‐SOFCs had the same support geometry and dimensions with MF‐SOFCs, differing only in material composition and pore architecture. Since fuel starvation is a critical SOFC failure mode and key reliability metric, controlled fuel compositions were designed to assess starvation tolerance [47, 48]. A standardized N*x*H*y* nomenclature was used to denote fuel mixtures, where N0H5 corresponds to pure humidified hydrogen as the baseline condition, N1H4 represents a 1:4 volume ratio mixture of N_2_ and H_2_ to induce initial fuel starvation, with progressively severe starvation conditions (N2H3 to N4H1) following this pattern.

**FIGURE 3:**
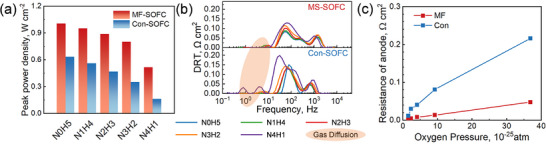
Electrochemical performance measured under different fuel compositions at 650°C. (a) Comparison of peak power densities between MF‐SOFC and Con‐SOFC. (b) DRT curves derived from EIS measurements of MF‐SOFC and Con‐SOFC. (c) Anode resistance as a function of oxygen pressure at the anode.

As evidenced by the peak power density comparison at 650°C (Figure 3a), the MF‐SOFC demonstrated superior performance with 1.01 W cm^−x^ ^2^ vs. 0.64 W cm^−^^2^ for the Con‐SOFC in a hydrogen atmosphere, representing a 1.6‐fold enhancement. Both cells showed performance degradation under fuel starvation, with the degradation worsening with depletion severity. However, the MF‐SOFC exhibited a much slower decay rate than the Con‐SOFC, revealing the conventional cells’ greater susceptibility to fuel starvation. Notably, the metal foam architecture showed enhanced starvation resilience, even under extreme conditions with hydrogen content as low as 19.4 vol%. To elucidate the modified support's impact on electrochemical processes, the measured electrochemical impedance spectroscopy (EIS) datum were transformed into distribution of relaxation time (DRT) profiles, as presented in Figure 3b. The DRT spectrum of the MF‐SOFC exhibited no discernible low‐frequency peaks, indicating a unique electrochemical behavior. Specifically, the metal foam support markedly reduced anode concentration polarization (as highlighted in the light‐orange region), which in turn decreased anode activation polarization. Moreover, the high‐porosity, large‐pore metal foam structure enhances fuel transport efficiency, thereby reducing reliance on high‐purity fuels. The dependence of anode resistance on oxygen partial pressure was further analyzed according to the method developed by Zhu et al., as shown in Figure 3c [49]. Both the MF‐SOFC and Con‐SOFC showed increasing anode resistance with rising oxygen partial pressure under open‐circuit conditions. However, the MF‐SOFC exhibited significantly lower anode resistance than the Con‐SOFC at equivalent oxygen partial pressures, with the difference becoming more pronounced at higher pressures. These results validate the DRT analysis and explain the MF‐SOFC's superior performance under fuel starvation compared to conventional designs.

### 2.2 Mechanism Underlying the High Specific Power of MF‐SOFCs

**FIGURE 4:**
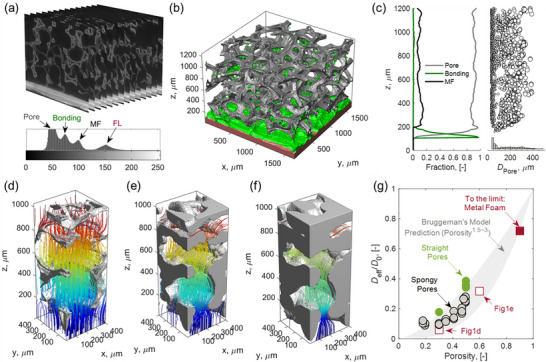
3D reconstruction and analysis of the MF‐SOFC. (a) 2D image series with corresponding grayscale distribution histograms used for phase segmentation of pores, bonding layer (Bonding), metal foam support (MF), and functional layer (FL). (b) Reconstructed 3D microstructure showing pores (transparent), metal foam (gray), bonding layer (green) and functional layer (red), respectively. (c) Volumetric distributions of pores, bonding layer, and metal foam, with pore diameter (*D*_pore_) distribution along the thickness direction (z‐axis). (d) Streamline visualizations of a generic field along the z‐direction in metal foam pores obtained by solving Laplace's equation, with field potential decreasing from red to blue. Simulated generic field distributions in pores with porosities of (e) 0.6 and (f) 0.3 through metal foam network dilation. (g) Relative gas diffusivity in the pores as a function of porosity, guided by the Bruggeman's model. Circle markers represent literature data for micropores and straight pores; square markers are calculated from field distributions in (d–f).

According to the tetrahedral framework of the materials, the differences in electrochemical performance between MF‐SOFCs and Con‐SOFCs can be attributed to their distinct microstructural characteristics, as revealed by 3D computed tomography (3D‐CT) analysis. Figure 4a presents the reconstructed 2D tomographic image slices and the corresponding grayscale histogram of the MF‐SOFC, where four distinct peaks emerge from the statistical analysis. Based on the principle that materials with higher atomic numbers exhibit greater X‐ray absorption and, consequently, higher grayscale values, different structural components can be identified. The high‐Z (atomic number) rare earth elements (Gd, Ce, La) present in the functional layers (abbreviated as “FL”) exhibit the highest grayscale values, corresponding to the distinct peak at the far right of the histogram. In contrast, the pores in the MF‐SOFC, which are infiltrated with epoxy resin (composed primarily of low‐Z elements: C, H, and O), display the lowest grayscale values, represented by the leftmost peak. The exceptionally high intensity of this left peak (partially truncated in the displayed range) indicates that this phase dominates the microstructure volume fraction—consistent with the MF‐SOFC's characteristic high porosity and large‐pore architecture. Although the bonding layer shares an identical composition with the metal foam, its grayscale values are intermediate between the metal foam and pore regions due to the presence of incorporated microporosity. This results in smooth peak transitions between the bonding layer, metal foam, and pore in the histogram, unlike the sharply isolated peak corresponding to the functional layers.

The 3D reconstruction (Figure 4b) employs color‐coding (gray for metal foam; green for bonding layer; red for functional layers; transparent for pores) to visualize the graded transition from cathode to the metal foam support. This color‐coded segmentation enables quantitative analysis of the volumetric fractions and pore size distribution along the thickness direction (z‐axis) for each component, as statistically demonstrated in Figure 4c. The 3D visualization more clearly reveals how the bonding layer gradually thins from complete anode coverage to discrete attachment points on foam struts, ultimately disappearing completely at a depth of 200 µm. The metal foam maintains its structural integrity with a consistent thickness of approximately 1 mm and 90% porosity after co‐sintering, showing negligible dimensional change from its initial state. Notably, the pore size distribution exhibits gradual expansion along the z‐direction, ranging predominantly from 50 to 150 µm near the bonding layer to larger pores (150–400 µm) in the bulk metal foam.

The gas transport characteristics in supports with varying porosity were systematically investigated through numerical solutions of the Laplace equation. The results reveal the significant influence of the metal foam's unique porous structure on mass transport properties, as illustrated in Figure 4d–f. The simulated streamlines, color‐graded from red to blue, quantitatively represent the decreasing potential field distribution. At 90% porosity, the gas streamlines exhibit nearly straight trajectories with uniform distribution throughout the pore channels, achieving a near‐ideal tortuosity factor approaching 1. This structural configuration minimizes gas transport resistance, enabling efficient fuel delivery to the anode even under severe fuel starvation conditions. Consequently, this explains the substantially reduced support‐induced losses in MF‐SOFCs compared to Con‐SOFCs. When the porosity decreases to 60% (analogous to the straight‐pore structure in Figure 1e) and further to 30% (resembling the microporous structure in Figure 1d), the streamlines demonstrate both markedly increased tortuosity and significantly reduced density, resulting in dramatically elevated gas transport resistance and consequently greater support‐related polarization losses.

According to the Bruggeman model, increasing porosity enhances the effective gas diffusion coefficient, as demonstrated in Figure 4g. The gray shaded area represents the theoretical relationship between porosity and gas diffusivity for porous SOFC electrodes and supports. Experimental data from the literatures are plotted as green circles (straight‐pore architectures) and gray circles (microporous structures), while red open squares denote values obtained from streamline simulations, which show excellent agreement with experimental measurements [50, 51, 52, 53, 54, 55, 56, 57, 58, 59]. The red solid square corresponds to the metal foam support developed in this study, where the ultrahigh porosity of 90% achieves an exceptional effective diffusion coefficient of 0.75, representing approximately 2‐fold and 4‐fold improvements over straight‐pore and microporous configurations, respectively.

### 2.3 Mechanical Properties of MF‐SOFCs

**FIGURE 5:**
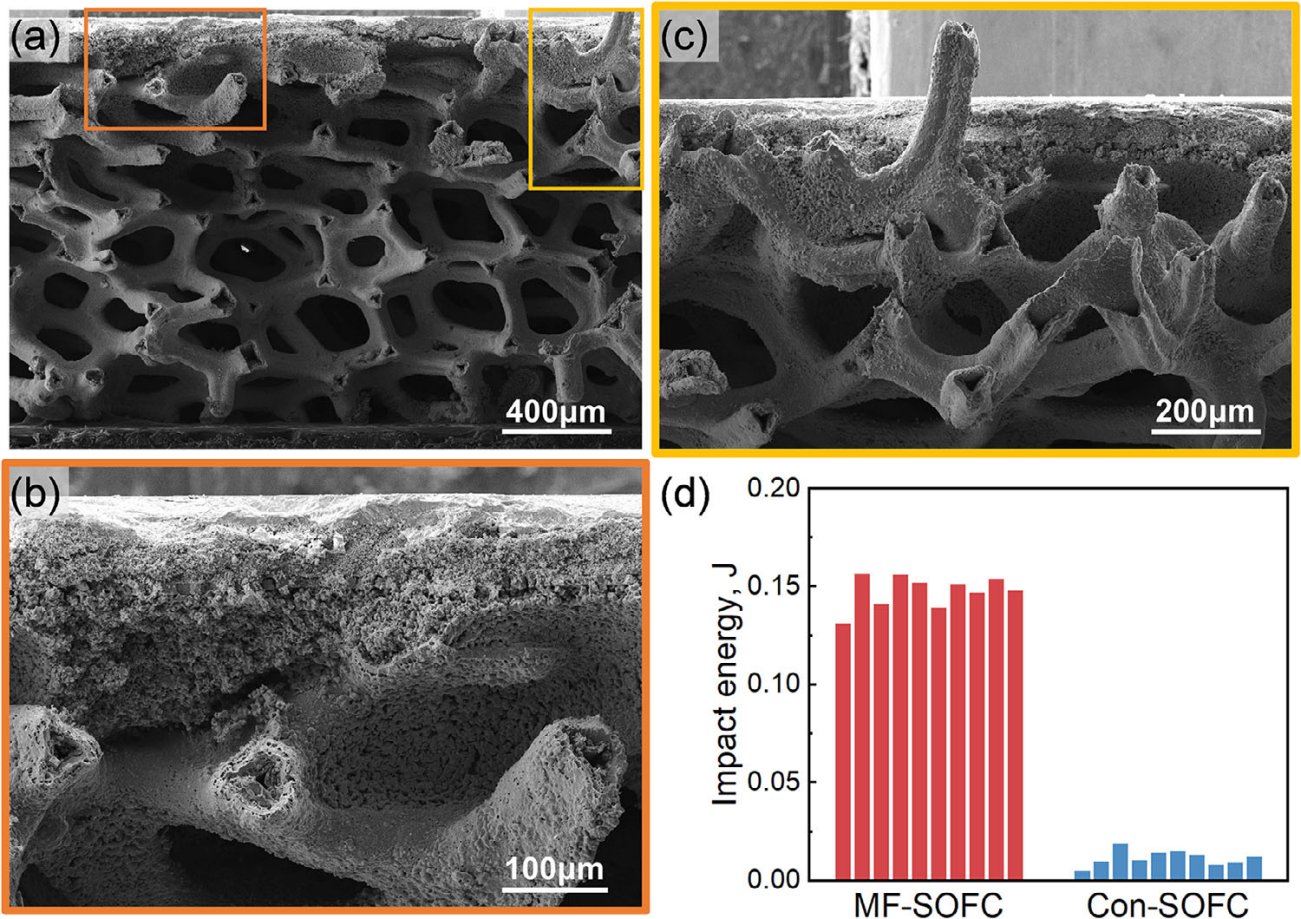
Cross‐sectional morphology of MF‐SOFC after impact fracture: (a) macroscopic fracture morphology of MF‐SOFC, (b) interface between anode functional layer and metal foam and (c) morphology of plastically deformed metal foam. (d) Comparison of impact toughness between MF‐SOFC and Con‐SOFC.

To evaluate the interfacial strength between the bonding layer, metal foam, and functional layers, impact tests were conducted to compare the impact toughness of MF‐SOFCs and Con‐SOFCs. Figure 5a presents the fracture morphology of the MF‐SOFC after impact testing. The metal foam and functional layers remained well‐bonded, with the functional layers retaining structural integrity and showing no significant delamination.

Figure 5b presents a high‐magnification image of the orange‐framed region in Figure 5a, specifically highlighting the interface between the anode and metal foam. While microcracks formed at the bonding layer/functional layer interface, the majority of the bonding area retained strong adhesion to the anode, with no macroscopic delamination. Figure 5c examines the yellow‐marked zone in Figure 5a in detail, clearly showing that the metal foam framework underwent plastic deformation along the impact direction. These findings confirm that the impact energy was efficiently transferred through the bonding layer and subsequently absorbed by the metal foam's plastic behavior, thereby preventing catastrophic failure.

The impact toughness values of MF‐SOFCs and Con‐SOFCs are presented in Figure 5d. The quantitative analysis reveals that MF‐SOFC exhibits a substantially higher average impact toughness of 0.148 J compared to just 0.012 J for Con‐SOFC, representing an order‐of‐magnitude improvement in mechanical resilience. This dramatic enhancement provides direct evidence of the superior impact resistance achieved in MF‐SOFC designs, resulting from the optimized interfacial bonding between the bonding layer, metal foam support, and functional layers. Statistical evaluation of the test data shows standard deviations of 0.008 and 0.004 for Con‐SOFC and MF‐SOFC, respectively. These relatively narrow distributions in measured values demonstrate excellent repeatability of the fabrication process and confirm the high consistency in mechanical performance across multiple samples. Together, the significantly enhanced impact toughness and tight performance distribution validate the superior reliability and scalable manufacturability of the MF‐SOFC design.

## 3 Conclusion

In this study, MF‐SOFCs were fabricated using an innovative approach that involved pre‐fabrication of integrated functional layer units, followed by their robust connection to metal foam supports via a transitional bonding layer compositionally matched to the metal foam. The resulting metal foam support exhibited exceptional structural characteristics, with 90% porosity and an average pore size of 100 µm. This methodology successfully enabled the realization of large‐pore metal foam‐supported SOFCs while preserving the intrinsic porous architecture of the metallic substrate, achieving a remarkable areal mass of only 0.15 g cm^−^^2^. These results represent a significant advancement in lightweight SOFC design.

The unique pore architecture of the metal foam support enables the effective gas diffusion coefficient to approach near‐theoretical limits. This remarkable feature substantially reduces gas transport resistance and effectively mitigates concentration polarization, thereby lowering anode resistance and preventing it from becoming the dominant limiting factor. Consequently, the MF‐SOFC demonstrates superior electrochemical performance and enhanced stability compared to Con‐SOFCs, even under fuel starvation conditions. When operated at 650°C in a humidified hydrogen atmosphere, the MF‐SOFC delivers a peak power density of 1.01 W cm^−^^2^ with a specific power of 6.56 kW kg^−1^. These performance metrics surpass those of conventional SOFCs with various architectures by factors ranging from several to dozens, offering a viable pathway for developing lightweight SOFC systems and broadening their practical applications.

## 4 Experimental Section

### 4.1 Fabrication of Single Cell

A metal foam‐supported SOFC single cell was fabricated using NiO, Gd_0.1_Ce_0.9_O_1.95_ (GDC), La_0.6_Sr_0.4_Co_0.2_Fe_0.8_O_3‐δ_ (LSCF), Fe and Ni powder (SOFCMAN, China), and Fe‐Ni metal foam. The anode/electrolyte bilayer was prepared by tape‐casting and co‐sintering. Initially, NiO and GDC powders in a 1:1 mass ratio were ball‐milled in ethanol with a dispersant for 24 h. The resultant slurry was further processed with binders and plasticizers for 4 h to form the anode slurry. The electrolyte slurry was prepared following an identical procedure but without NiO. Both slurries were tape‐cast onto a substrate, dried, and co‐pressed at 200 mPa before being co‐sintering at 1500°C for 4 h. Subsequently, a cathode ink (50 wt.% LSCF‐GDC) was screen‐printed onto the bilayer and sintered at 1050°C for 2 h to form a tri‐layer structure. Finally, the Fe‐Ni metal foam was bonded to the tri‐layer using a Fe‐Ni slurry (with identical composition to the metal foam) and sintered at 950°C for 2 h under Ar atmosphere. For comparison, a conventional metal‐ceramic composite anode‐supported cell (denoted as Con‐SOFC) was fabricated identically, maintaining consistent layer thicknesses.

### 4.2 Microstructural Characterization and Mechanical Property Evaluation

Microstructural analysis was performed using a scanning electron microscopy (SEM, SUPRA55, ZEISS) with an accelerating voltage of 20 kV, while 3D morphology was obtained via 3D computed tomography (3D‐CT, Xradia 520 Versa, ZEISS). The 2D cross‐sectional samples were prepared for observation by first embedding them in epoxy resin (Epo‐Fix) to infiltrate the pores within the SOFCs, followed by grinding and polishing of the cured samples. Due to the small dimensions of the SOFC samples, conventional standardized testing methods were impractical. Therefore, a custom impact testing apparatus was developed based on the same fundamental principles to evaluate impact toughness. This property was calculated by measuring the maximum height reached by the pendulum after its release from a horizontal position and subsequent fracture of the specimen.

### 4.3 Electrochemical Characterization

Electrochemical performance was evaluated using a custom test rig with a Zahner Zennium E workstation. *V*‐*I* curves were obtained by linear sweep voltammetry, with the voltage scanned from the open‐circuit voltage (OCV) to 0.2 V at increments of 0.01 V. The inflow rate of the humified N_2_‐H_2_ gas mixture was fixed at 20 mL/min. The electrochemical impedance spectroscopy was performed over a frequency range of 1 mHz to 0.1 Hz and analyzed using the distribution of relaxation time method.

### 4.4 Data Analysis

The cell‐level specific power was calculated by dividing the areal peak power density by the corresponding areal mass. All reported specific power values are derived from the direct calculation of the original raw data, rather than from processed figures that have been rounded in the main text for brevity and clarity.

## Conflicts of Interest

The authors declare no conflicts of interest.

## Supporting information




**Supporting File**: advs74022‐sup‐0001‐SuppMat.docx.

## Data Availability

The data that support the findings of this study are available from the corresponding author upon reasonable request.
