# Supplementary material for: A Novel Metal Foam‐Supported Solid Oxide Fuel Cell With High Specific Power
Source: Adv Sci (Weinh). 2026 Feb 12;13(19):e17694. doi: 10.1002/advs.202517694 (PMC13045212; doi:10.1002/advs.202517694)
Supplement: Supplementary file 1 — Supporting File: advs74022‐sup‐0001‐SuppMat.docx. [file ADVS-13-e17694-s001.docx]

Supporting Information

A Novel Metal Foam-Supported Solid Oxide Fuel Cell with High Specific Power

Jingbo Ma^1,2^, Ying Yang^1^, Meng Ni^3^, Yanxiang Zhang^1^*, Mufu Yan^1^*

^1.^ School of Materials Science and Engineering, Harbin Institute of Technology, Harbin 150001, China

^2.^ China Academy of Machinery Beijing Research Institute of Mechanical & Electrical Technology Co.,Ltd., Beijing 100083, China

^3.^Department of Building Environment and Energy Engineering, Research Institute for Sustainable Urban Development & Research Institute for Smart Energy, The Hong Kong Polytechnic University, Hung Hom, Kowloon, Hong Kong 999077, China

**Figure S1.** Comparison of specific power versus areal peak power density at different temperatures between the present work (represented by red five-pointed stars) and literature-reported SOFCs, including H-SOFCs (represented by blue symbols), cermet-supported O-SOFCs (represented by green symbols), and metal-supported O-SOFCs (represented by purple symbols).

(a) 500 °C, (b) 550 °C, (c) 600 °C, (d) 650 °C


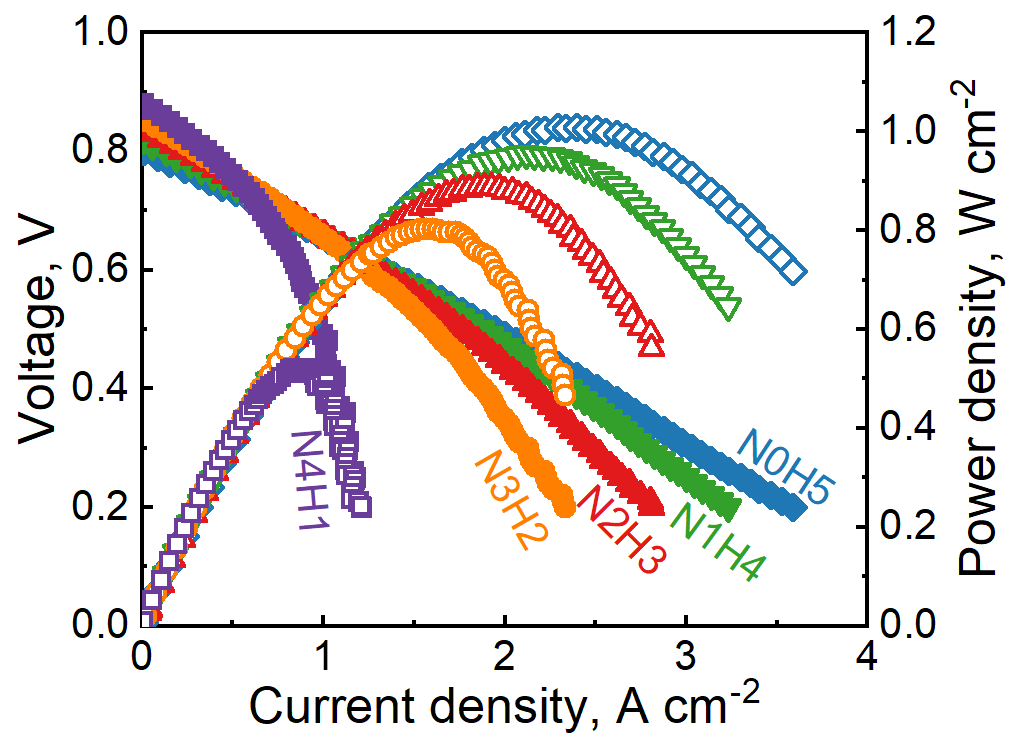


**Figure S2.** *I*–*V* and *I*–*P* curves of MF-SOFC at 650 °C under humidified (3 vol% H_2_O) fuel atmospheres with varying N_2_:H_2_ ratios.

**Figure S3.** DRT of MF-SOFCs and Con-SOFCs measured under open-circuit conditions at (a) 500 °C, (b) 550 °C and (c) 600 °C.

**Figure S4.** Multi-physics modeling and analysis of internal losses at 650 °C of the MF-SOFC and Con-SOFC.

(a) Experimental and simulated *I*–*V* curves. (b-d) Comparison of internal losses of (b) support, (c) cathode and (d) anode under various fuel atmospheres.


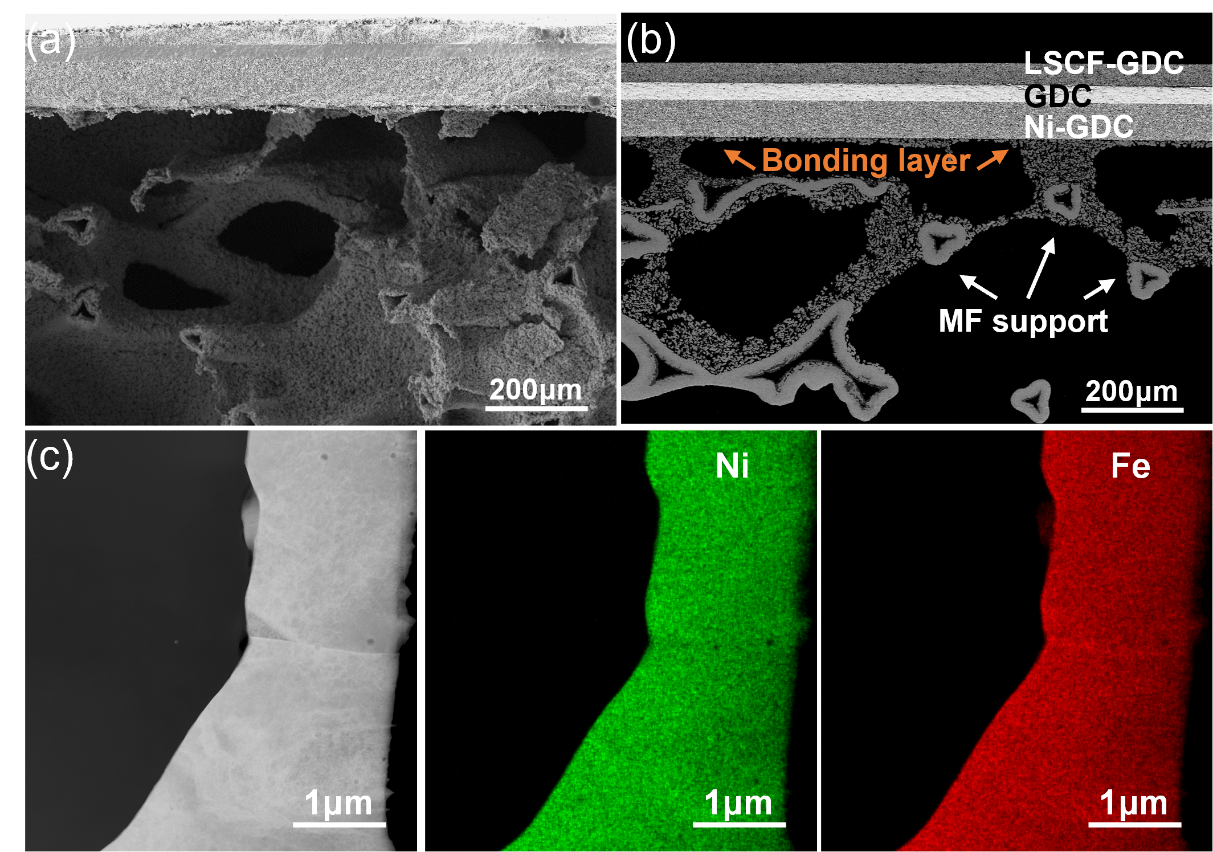


**Figure S5.** Microstructural characterization and elemental analysis of the MF-SOFC.

(a) Fracture cross-sectional morphology. (b) 2D cross-sectional morphology of epoxy-infiltrated MF-SOFC. (c) HAADF-STEM image of the bonding layer/metal foam interface with corresponding Ni and Fe elemental maps.
